# Complex interactions among invasive fishes and flow barriers combine to maintain an endangered native species

**DOI:** 10.1007/s10530-026-03935-y

**Published:** 2026-09-24

**Authors:** Danai Kontou, Dumisani Khosa, Sunčica Avlijaš, Anthony Ricciardi, Nicholas E. Mandrak, Olaf L. F. Weyl, Josie South

**Affiliations:** 1 Water@Leeds, School of Biology, Faculty of Health and Life Sciences, University of Leeds, Leeds, LS2 9JT UK; 2 Scientific Services, South African National Parks, Skukuza, 1350 South Africa; 3 South African Institute for Aquatic Biodiversity, Makhanda, 6139 South Africa; 4 Fisheries and Oceans Canada, Bedford Institute of Oceanography, Dartmouth, NS B2Y 4A2 Canada; 5 Department of Biology, McGill University, Montreal, QC H3A 1B1 Canada; 6 University of Toronto, Toronto, ON M1C 1A4 Canada; 7 Centre for Invasion Biology, Faculty of Health and Life Sciences, University of Leeds, Leeds, LS2 9JT UK

**Keywords:** Invasive species, Freshwater ecology, Fish conservation, Human footprint, Species interactions

## Abstract

The Breede River in the Western Cape Province is one of South Africa’s major freshwater systems and of high ecological and socio-economic value. Similar to other freshwater systems worldwide, it is threatened by environmental degradation and multiple invasive species. Understanding the biotic and abiotic processes that facilitate or hinder establishment and impact of co-occurring biological invasions is a critical knowledge gap that prevents proactive management efforts. This study combined data from an extensive monitoring survey with indices of human impact to explore whether biodiversity patterns indicate signals of environmental filtering and invasion meltdown using freshwater fish diversity and abiotic factors along the entire length of the Breede River. Native fish species occupancy was uneven while invasive non-native fishes were widely distributed and represented over 40% of the overall species richness. Fish diversity was negatively correlated with the relative frequency of a translocated predator, the sharptooth catfish (*Clarias gariepinus*) but positively correlated with the frequencies of invasive black basses (*Micropterus nigricans, M. dolomieu*). Flow barriers, high abundance of competitive catfish, and increased water turbidity suppressed black bass predation and influenced fish-community dynamics, allowing an endangered native species, the Berg-Breede whitefish (*Cheilobarbus capensis*), to persist at extremely low frequencies. Thereby, we show empirically that the ecosystem engineering behaviours of the turbidity-adapted sharptooth catfish suppress the high predatory impact of visual predators, black basses. Our results show that the Breede River is heavily impacted by species invasions, but that these species have antagonistic interactions which moderate ecological impact.

## Introduction

The accelerating rate of environmental change, from intensifying land use to damaging non-native species introductions, puts wildlife under immense pressure and is reshaping entire ecosystems (Pörtner et al. 2023). Freshwater habitats are particularly susceptible to multiple co-occurring environmental stressors that drive biodiversity declines and threaten their integrity (Birk et al. 2020; Tickner et al. 2020). Invasive non-native species are a major stressor and one of the leading causes of biodiversity loss worldwide (Roy et al. 2024). Fishes are one of the taxa most highly impacted by species invasions, especially in fresh waters (Cavalcante et al. 2025). At the same time, the widespread establishment of freshwater fishes outside of their native ranges can have catastrophic consequences through various impact mechanisms e.g. predation, grazing, competition, hybridisation (Britton 2023; Carneiro et al. 2025). Ecological impacts can cascade across impact types, and understanding the interactions between typologies (i.e., the consequences of the impacts) is crucial to fully capture changes caused by biological invasions on both the biotic and abiotic aspects of a system (Carneiro et al. 2025). However, increasing complexity arises when faced with untangling the outcomes from multiple biotic and abiotic drivers of change.

In systems facing multiple species invasions, it is hypothesised that facilitative interactions among non-native species can generate greater invasion rates and risk of synergistic disruption–a process termed ‘invasional meltdown’ (Simberloff and Von Holle 1999; Ricciardi and Simberloff 2025). For example, in the Laurentian Great Lakes, the ecosystem engineering capacity of non-native dreissenid mussels supports native and non-native invertebrate populations, thus providing abundant prey for a non-native benthivorous fish, the round goby (*Neogobius melanostomus*) (Ricciardi 2001). Alternatively, non-native species can interfere or suppress each other in ways that dampen their respective impacts (e.g., Griffen et al. 2008; Van Riel et al. 2009). Therefore, beyond individual species impacts, interactions between sympatric invaders can have complex antagonistic, synergistic, or cumulative effects that are significant to conservation but may be difficult to both predict and manage (Ahmad et al. 2025; Braga et al. 2020; Jackson 2015; Preston et al. 2012; Ricciardi & Simberloff 2025). In dynamic environments such as rivers (Brauns et al. 2022; Harris and Heathwaite 2012), habitat structure, water chemistry, and complicated natural processes can further influence species interactions and invasion success (Barrios-O’Neill et al. 2015; Khosa et al. 2021; O’Briain et al. 2023; Woodford et al. 2024).

South African watercourses are fertile grounds for testing hypotheses in invasion science due to the proliferation of non-native invasive species and translocated extralimital species, most having been introduced by inter-basin transfer, recreational fishing, aquaculture, or bio-control pathways (Ellender and Weyl 2014; Khosa et al. 2019; Marr et al. 2017; South et al. 2022; van Wilgen et al. 2022). The Breede River in the Western Cape province has been invaded by non-native common carp (*Cyprinus carpio*), largemouth bass (*Micropterus nigricans)* and smallmouth bass (*M. dolomieu)* and the extralimital sharptooth catfish (*Clarias gariepinus*) and banded tilapia (*Tilapia sparrmanii*), among others (Ellender et al. 2011, 2015; Ellender and Weyl 2014; Khosa et al. 2019). *Cyprinus carpio*, introduced into the Western Cape in the 1800’s, has been widely established across South Africa since 1967. *Micropterus* spp. have been present in the Breede River since 1933, after the stocking of *M. nigricans* fingerlings in the Brandvlei Dam; however, more recently, it appears that most black bass in the Breede River are hybrids between *M. nigricans* and the Florida bass, *M. floridanus* (Khosa et al. 2019). *Clarias gariepinus* is a more recent invader after its direct introduction in 1999 (Weyl et al. 2016). Using a priori knowledge of invasion dynamics, we examined whether patterns were consistent with predictions of invasion meltdown or environmental filtering, in which we would expect a positive relationship between *Cyprinus carpio* and *Clarias gariepinus,* benthivorous species with compatible habitat preferences*,* as *C. carpio* forage by grubbing and sediment disturbance, which increases turbidity, and *C. gariepinus* is a generalist adapted to highly turbid water. Conversely, black basses are visual pursuit piscivores (Taylor et al. 2019; Luger et al. 2020) and would be limited by highly turbid water.

The Breede River, with its tributaries and surrounding valley, are of high ecological and socio-economic value, providing critical freshwater habitat (DWAF 2003) and supporting local economies (agriculture, tourism, recreational fishing) (Cullis et al. 2018). Healthy and stable ecosystems rely on diverse species communities and their functions (Lynch et al. 2023), monitoring diversity on multiple levels is essential for effective management and conservation (Tickner et al. 2020). Despite the indisputable value of freshwater resources and global efforts monitoring invasive species, large-scale biodiversity assessments of entire waterbodies are still limited (Altermatt et al. 2020; Reid et al. 2019). We use a unique, standardised source-to-sea fish survey to address key questions, such as 1) whether interactions between co-occurring invasive species are facilitative (consistent with invasional meltdown processes) or antagonistic; 2) if environmental filtering is acting upon the fish community; and, 3) how the establishment of multiple non-native species have re-shaped fish communities along the entire length of the Breede River. Our overarching aim was to use theory from invasion and community ecology to understand the key drivers behind changes in river fish-community dynamics from source-to-sea, and to gain an insight into which factors might have allowed non-native fishes to successfully establish in portions of the Breede River, their interactions, and their impacts on native species distribution.

## Materials & methods

### Study area

**Fig. 1:**
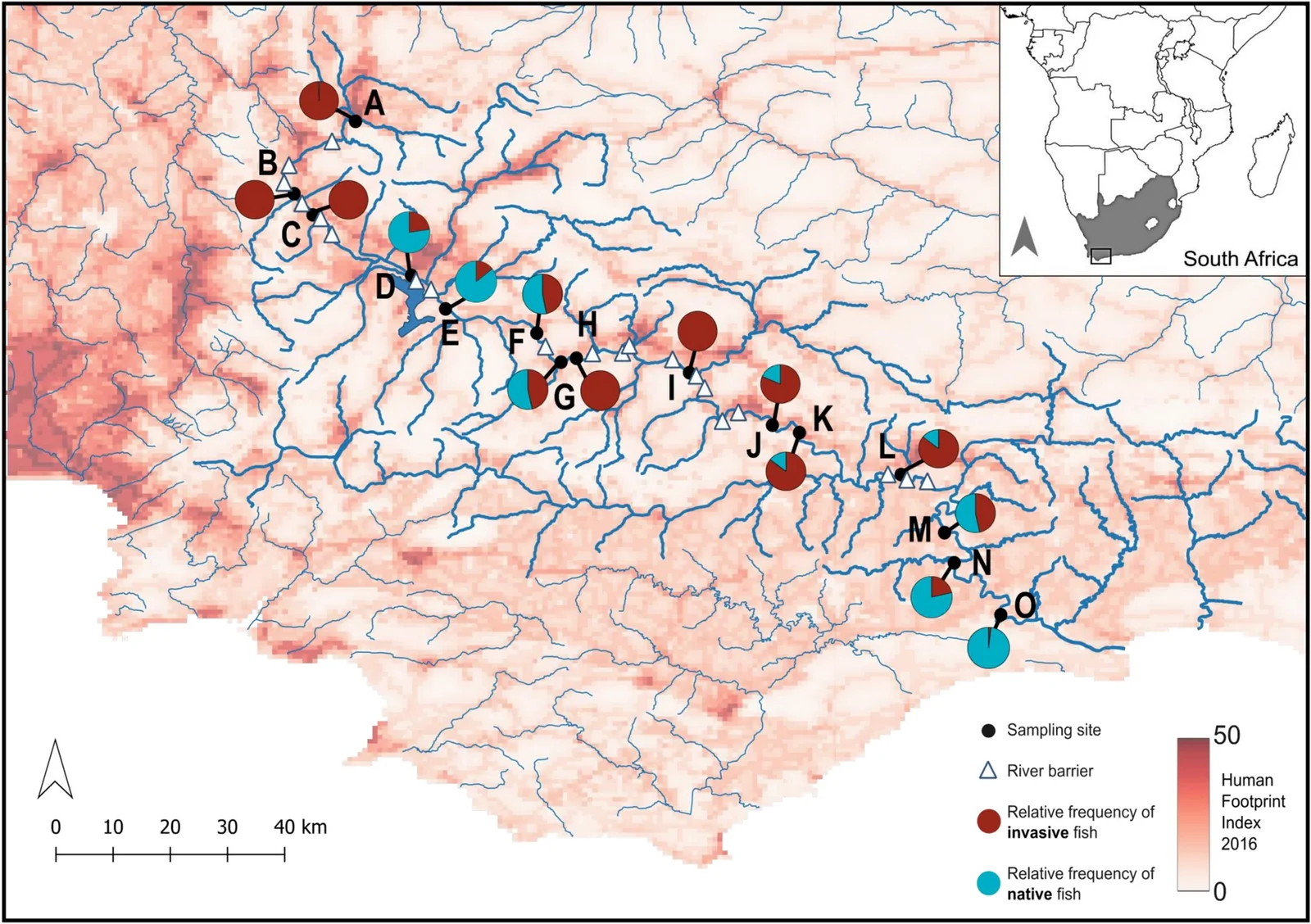
Breede River Valley, Western Cape, South Africa. The lines in bold highlight the mainstem section of the river and its tributaries. The additional layer of the cumulative Human Footprint Index data for the year of sampling (2016), was calculated based on human population density and land use intensity for infrastructure, pasture, and agriculture (Mu et al. 2022). Pie charts represent the relative proportion of native (blue) versus non-native (red) fishes caught in each sampling site (black), ordered alphabetically from source (A) to sea (O). Site (D) marks the location of the Greater Brandvlei Dam irrigation lake which connects to the Breede River. Triangles mark locations of flow barriers such as weirs, that control water flow and can restrict fish movement along the river

The Breede River drains a catchment area of ~12,300 km^2^ in the Western Cape of South Africa and flows towards the south-east for approximately 320 km from its source in the Ceres Mountains (Skurweberg) to its mouth at the Indian Ocean, forming a large estuary near Witsand. It is joined by several tributaries, and there are multiple weirs serving as flow barriers along its length, as well as dams in surrounding waterbodies. The Greater Brandvlei Dam and adjacent Quaggaskloof Dam are located in the upper catchment of the river and since 1972, they form a single reservoir that serves as an irrigation lake with its outflow in the main river body (DWAF 2003). A total of 15 sites covering the entire length of the river’s mainstem section were sampled between October and December 2016, moving upstream from the estuary (BR01/A) to the source (BR16/O) (Table 1; Fig. 1). To this day, it remains the only comprehensive survey of the Breede mainstem, and the data collected in 2016 are the only available baseline of the river’s fish community.

**Table 1:** Breede River 2016 sampling-effort summary. Sampling location coordinates, site names and alphabetic codes used for analyses

Latitude	Longitude	Site code		Fyke			Gill			Seine		
				Nets	Fish	Spp	Nets	Fish	Spp	Nets	Fish	Spp
−34.3359	20.6042	BR01	A	6	6	5	5	11	4	6	168	4
−34.2356	20.5112	BR02	B	6	3	2	5	20	4	6	121	6
−34.1774	20.4918	BR03	C	6	6	3	5	36	6	6	3	2
−34.0712	20.4057	BR05	D	6	17	3	5	45	5	6	45	5
−33.9843	20.2017	BR07	E	6	6	1	5	57	5	6	14	2
−33.9698	20.1476	BR06	F	6	6	2	5	20	5	6	19	3
−33.8700	19.9871	BR08	G	6	94	3	5	16	2	6	36	2
−33.8405	19.7508	BR09	H	6	11	1	5	3	1	6	149	3
−33.8480	19.7250	BR11	I	6	6	1	5	14	4	6	23	1
−33.7920	19.6765	BR10	J	6	13	3	5	12	3	6	86	3
−33.7452	19.4941	BR12	K	6	15	2	5	15	3	6	233	4
−33.6872	19.4278	BR13	L	6	29	3	5	40	4	6	209	4
−33.5661	19.2302	BR14	M	6	48	2	5	17	4	6	193	3
−33.5228	19.1914	BR15	N	6	19	1	5	13	3	6	3	3
−33.3830	19.3141	BR16	O	6	1	1	5	1	1	6	644	2
		Total fish			280			320			1946	
		Unique species				11			13			12
		Total nets		90			75			90		

Number of nets deployed at each site (Nets), number of fish caught (Fish) and number of uniquely identified species (Spp) per site and net type (fyke, gill, seine)

### Fish survey and habitat data

A standardised multi-method approach was used to collect fish under a Cape Nature permit Ref. 0056-AAA04100155. A combination of six seine nets (5 × 2 m long; 1.5 m deep; 5 mm mesh size), six double-ended fyke nets (8 m guiding net; 55 cm first ring diameter; 10 mm mesh size) and five gillnets (35 m long; 2.75 m deep) of differing mesh sizes (35, 45, 57, 73, 93, 118, 150 mm) were deployed at each site to maximise area coverage based on habitat availability (Table 1). Collected fish were identified to species level and classified as either native or non-native to the Breede River (Skelton 2001). In every net, up to thirty fish of each species were measured for total length to the nearest millimetre (mm). Environmental parameters recorded at three locations in each site included: vegetation coverage (%); water flow category; sediment type (estimated visually using the Wentworth scale (1922)) and water turbidity ~20 cm below the surface (nephelometric turbidity units**(**NTU), using a LaMotte 2020we Turbidity meter); and dissolved oxygen (DO mg L^−1^), water temperature (°C) and specific conductivity, measured using a YSI Pro 2030 multiprobe with a 3 m long cable at surface and bottom, with values averaged for analyses. Site elevation (metres above sea level) and visible flow barrier positions were extracted based on sampling coordinates (Table 1) with Google Earth Engine v. 10.87.0.1 (Satellite Imagery of Breede River Valley available via Google Earth; accessed March 2025).

Human Footprint data for the Province of Western Cape, South Africa, in the year of sampling (2016) were retrieved from published datasets by Mu et al. (2022). The cumulative Human Footprint Index (HFPI) is used as a measure of human pressure on natural environments and combines land coverage data for human infrastructure, population density, and agricultural land. The dataset used in this study is scaled from an index of 1 to 4 for intact wilderness, and 4 to 50 for intense land modification according to the framework presented in Mu et al. (2022).

### Fish species and size class distributions

All data analysis was completed in R v.4.4.2 (R Core Team 2025).

Sampling effort was standardised across sites (Table 1), therefore, to compare the diversity of the fish community along the river, we calculated the relative frequency of individual fish species, the proportion of native versus invasive fish and the Shannon Diversity Index. Shannon Diversity Index was calculated for each sampling site with the *diversity* function from the R package vegan v.2.8 (Oksanen 2015). For the purposes of this study, *C. gariepinus* and *T. sparrmanii* were grouped together with the other invasive species (Table 2). However, it is important to note that *T. sparrmanii* and *C. gariepinus* are native to other parts of South Africa but extralimital to the Breede River system (Skelton 2001; Weyl et al. 2016).

**Table 2:** List of recorded fish species, respective families, common names, and their classification as native or invasive in the Breede River catchment

Family	Species	Common names	IUCN status	
Anguillidae	*Anguilla mossambica* (Peters, 1852)	African longfin eel	NT	Native
Anabantidae	*Sandelia capensis* (Cuvier, 1831)	Cape kurper	DD	Native
Centrarchidae	*Lepomis macrochirus* Rafinesque, 1819	Bluegill sunfish	LC	Invasive
	*Micropterus dolomieu* (Lacepède, 1802)	Smallmouth bass	LC	Invasive
	*Micropterus nigricans* (Cuvier, 1828)	Largemouth bass	LC	Invasive
Cichlidae	*Tilapia sparrmanii* Smith, 1840	Banded tilapia	LC	Invasive*
Clariidae	*Clarias gariepinus* (Burchell, 1822)	Sharptooth catfish	LC	Invasive*
Cyprinidae	*Cyprinus carpio* Linnaeus, 1758	Common carp	VU	Invasive
	*Cheilobarbus capensis* (Smith, 1841)	Berg-Breede whitefish	EN	Native
Ehiravidae	*Gilchrestella aestuaria* (Gilchrist, 1913)	Estuarine round herring	LC	Native
Gobidae	*Redigobius dewaali* (Weber, 1897)	Checkered goby	LC	Native
Haemulidae	*Pomadasys commersonii* (Lacepède, 1801)	Spotted grunter	LC	Native
Monodactylidae	*Monodactylus argenteus* (Linnaeus, 1758)	Silver/Oval moony	LC	Native
	*Monodactylus falciformis* Lacepède, 1801	Round/Cape moony	LC	Native
Mugilidae	*Mugil cephalus* Linnaeus, 1758	Flathead mullet	LC	Native
	*Myxus capensis* (Valenciennes, 1836)	Freshwater mullet	LC	Native
Tincidae	*Tinca tinca* (Linnaeus, 1758)	Tench	LC	Invasive

IUCN status refers to species current status according to the most recent available assessment (IUCN Red List of Threatened Species): NT, near threatened; DD, data deficient; LC, least concern; VU, vulnerable; EN, endangered

^*^The African sharptooth catfish and banded tilapia are both extralimital to the Breede River but native in other South African catchments

To compare distributions of fish lengths, individuals were binned into the following size classes and plotted across sites to facilitate comparisons: (1) <50 mm (2) 50–150 mm; (3)151–250 mm; (4) 251–350 mm; (5) 351–450 mm; (6) 451–550 mm; and (7) >550 mm.

To visualise the spatial distribution of sampling sites, invasive species presence and human pressure including artificial flow barriers along the river length, we mapped species relative frequencies with the cumulative HFPI data for 2016 (Mu et al. 2022) using the open-source software QGIS v.3.34.15 (www.qgis.org) and regional shapefiles retrieved from the Department of Water and Sanitation, South Africa. Mean values of HFPI were calculated and extracted for an area with a close radius of 10 km around each site using the QGIS Zonal Statistics Plugin (QGIS Development Team 2025).

### Statistical analyses

We performed chi-squared tests of independence to determine the relationship between observed species diversity along the river and the relative frequency of the three most widespread introduced fish predators *C. gariepinus, M. nigricans,* and *M. dolomieu*. Due to low overall densities of the two bass species, *M. nigricans* and *M. dolomieu*, we also estimated a combined bass relative frequency for each site. In both cases, the Shannon Diversity Index was re-calculated to exclude the respective invasive species and accurately assess their impact. *p*-values were simulated based on 2000 replicates to account for small sample sizes. Additional chi-square tests were performed to test for associations between fish size classes and assess whether invasive predator interactions and impacts on native prey are driven by gape limitation. We examined the relationships between different size classes of three introduced piscivores (*C. gariepinus*, *M. nigricans*, *M. dolomieu)*, an introduced omnivore (*C. carpio*), and an endangered native prey species (*Cheilobarbus capensis*), measured at the time of sampling.

To assess whether the pattern of species presence/absence and overall distribution is determined by local habitat characteristics (elevation, vegetation coverage, water depth, turbidity, specific conductivity, flow category, temperature and dissolved oxygen), we conducted a canonical correspondence analysis (CCA) (ter Braak 1986) using the *vegan::cca* function (Oksanen 2015). Environmental variables were screened for autocorrelation by comparing variance inflation factors (VIFs), and any variables with VIF >10 were excluded from the final analysis. CCA relationships between species distribution and local habitat were evaluated with a Monte Carlo test (999 permutations) and a statistical significance level of *p* = 0.05.

We fitted generalised linear models (GLMs) to evaluate the effects of key environmental parameters (water turbidity, vegetation percent, mean human footprint index) and the relative frequencies of the highest-density invaders (*C. gariepinus, C. carpio, M. nigricans, M. dolomieu*) on the native species frequency along the river length. All models were fitted with the *glm* function using a Gamma distribution to account for right skewed positive frequency data with an added coefficient of +0.01 to correct for zero values. Stepwise model selection was conducted by generating an analysis of deviance table with the function *anova* and removing the variable with the highest *p*-value after each step. Variables with *p* < 0.1 were retained in the final model and only variables with a *p* < 0.05 were considered statistically significant predictors of native species relative frequency. To further assess which factors are driving low bass frequency, we fitted GLMs including the same environmental parameters and the relative frequencies of high-density invaders *C. gariepinus* and *C. carpio* as predictors of the log-transformed bass relative frequency. Stepwise model selection was conducted as described above.

## Results

### Changes in fish community along the river from source to sea

**Fig. 2:**
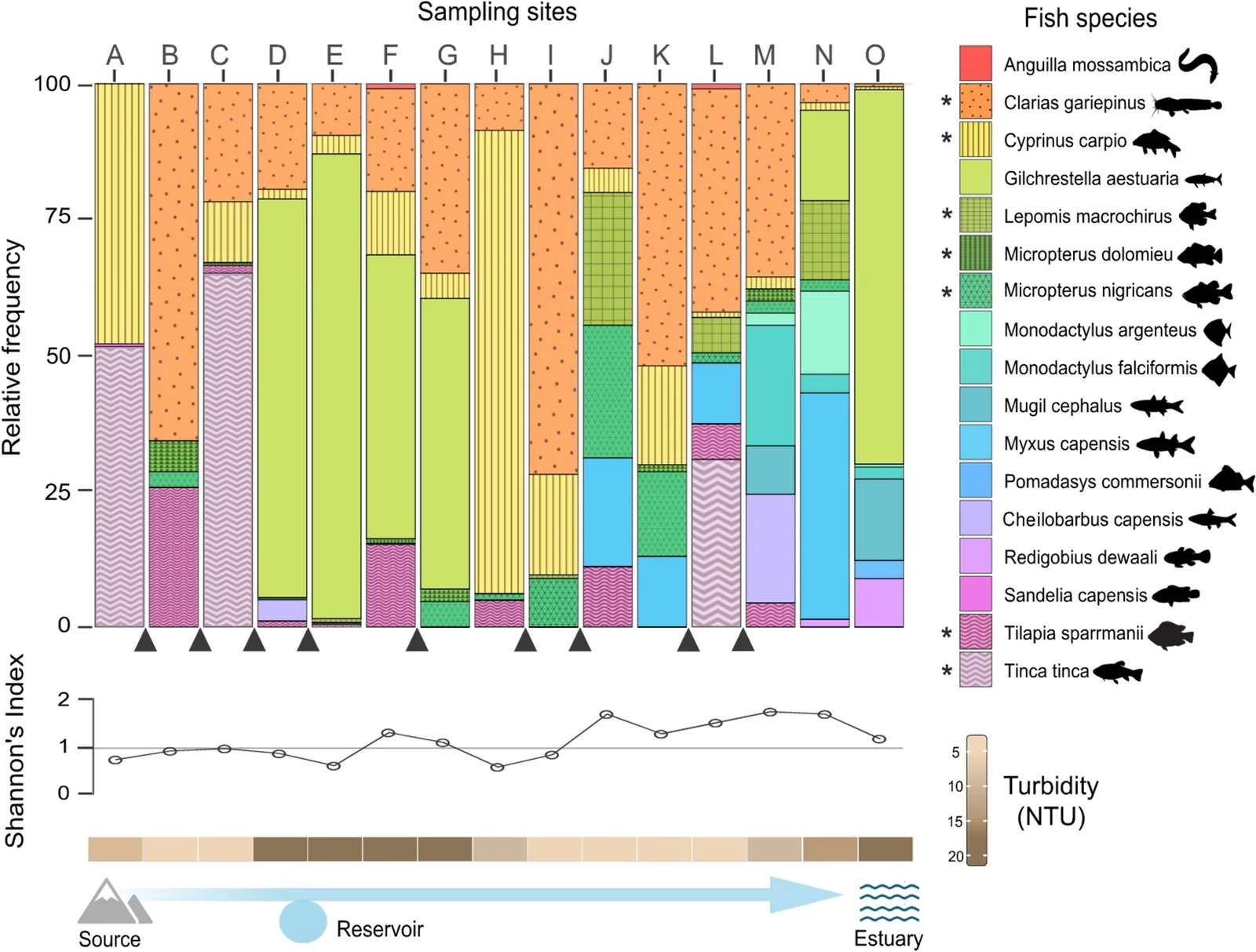
Species distribution and diversity changes along the length of the river, from source to sea, influenced by water turbidity and the presence of flow barriers. Each bar represents an individual sampling site, and each colour is proportionate to the relative frequency of a different fish species. Invasive species are marked with an asterisk in legend. Triangles between bars mark parts of the river where at least one visible flow barrier is present between sites. Line graph shows Shannon Diversity Index changes between sites and along the full length of the river. Bottom scale bar reflects changes in water surface turbidity measured at the time of sampling (nephelometric turbidity units—NTU). Arrow marks the direction of flow from the mountain source (A) to the estuary (O). Circle marks the location of the Greater Brandvlei Dam and reservoir

The survey covered the entire length of the mainstem Breede River and resulted in a total of 2546 fishes identified, of which 1609 were individually measured (Fig. 1; Table 1). There were 17 recorded fish species representing 12 families, of which seven were identified as introduced non-natives, corresponding to 41.2% of the overall species richness (Fig. 2; Table 2). At least two non-native fish species were present at each site but for sampling locations closer to the river’s source, the proportion of invasive species was close or equal to 100% (Figs. 1,2: sites A–C). In contrast, the three sites closest to the estuary were among the most diverse with very low frequencies of invasive fish species (Figs. 1, 2: sites M–O).

Native fish records included three species of concern: the endangered Berg-Breede whitefish, *Cheilobarbus capensis* (Impson et al. 2017), the near-threatened longfin eel, *Anguilla mossambica* (Pike et al. 2018), and the declining, but data-deficient, Cape kurper, *Sandelia capensis* (Chakona 2018) (Fig. 2; Table 2). Other native species, in higher frequencies (> 5%), included the estuarine round herring, *Gilchrestella aestuaria*, moonfishes, *Monodactylus* spp., freshwater mullets *Myxus capensis* and *Mugil cephalus*, spotted grunter, *Pomadasys commersonii* and the checkered gobby, *Redigobius dewaali* (Fig. 2). Invasive fish records included both predators and grazers, belonging to the following species and listed here in order of highest to lowest abundance: African sharptooth catfish, *Clarias gariepinus*, common carp, *Cyprinus carpio*, banded tilapia, *Tilapia sparrmanii*, bluegill, *Lepomis macrochirus*, largemouth bass, *Micropterus nigricans*, smallmouth bass, *Micropterus dolomieu* and tench, *Tinca tinca* (Fig. 2; Table 2).

As anticipated, the distribution of fish species along the river was not even. The most abundant native species were the estuarine round herring *G. aestuaria*, present downstream of the Brandvlei Dam reservoir lake (D–G) and near the estuary (N–O), and the freshwater mullet *M. capensis*, at the lower reaches of the river (J–O) (Fig. 2). *C. capensis* and *A. mossambica* were each limited to two distinct river sites (D/M and F/L respectively), while *S. capensis* was restricted to a single high-altitude site (A) and was the only native species occurring near the source of the river (Fig. 2). Extralimital *C. gariepinus* was absent near the source (A) but otherwise dominated the entire system, irrespective of flow barriers (Fig. 1) and with frequency decreasing only towards the estuary (N–O) (Fig. 2). Invasive *C. carpio* was the second most dominant species, reaching its highest frequency in the middle parts of the river (H) and near the source (A). The frequencies of both invasive bass species were low throughout the system. Similar to *C. gariepinus*, neither species was found near the source (A). However, bass species were also notably absent around the reservoir (D–E) (Fig. 2).

### Predictors of invasion pressure and impacts of invasive fish

Species distribution patterns along the Breede River were driven by physical barriers, predation, interspecific competition, and environmental parameters. The ubiquitous *C. carpio* was only absent from one site (B) near the source and between close upstream and downstream barriers (Fig. 1, 2). Other invasive species, such as *T. sparrmanii* and *T. tinca,* had patchy distributions with *T. tinca* generally restricted between barriers, especially in the lower parts of the river (L) (Figs. 1, 2). Endangered *C. capensis* persists exclusively in two sites with comparatively low predator abundances (D/M) (Fig. 2). Across all sites, we observed a significant negative relationship between the Shannon Diversity Index and the widely distributed *C. gariepinus* (X^2^ = 81.99, *p* < *0.01*) and a significant positive relationship with bass relative abundance (X^2^ = 20.33, *p* < *0.05*) (Fig. 4A, B).

**Fig. 3:**
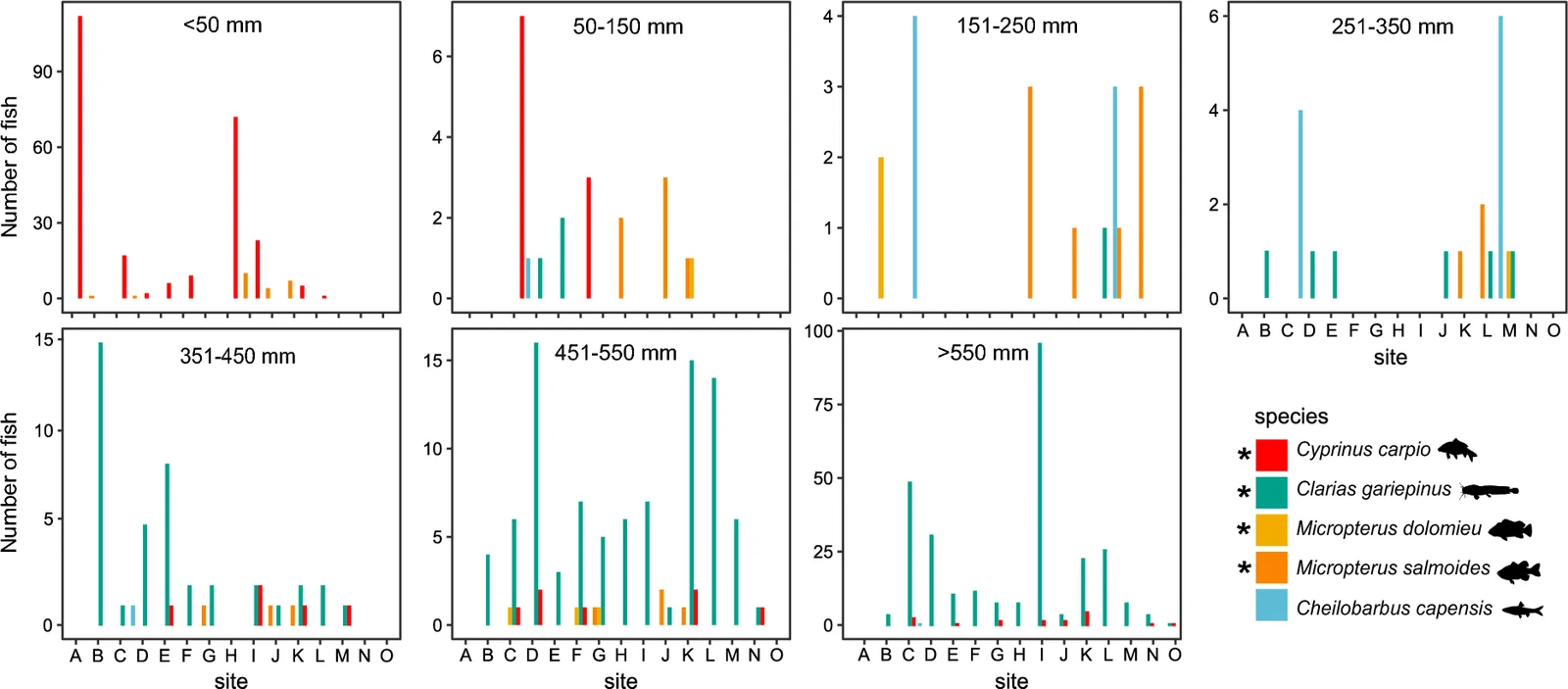
Significant size overlap of invasive predators, grazers and native prey reduces the impacts of gape limited predation and interspecific competition. Fish lengths (mm) are grouped in seven size classes to facilitate comparisons. Site order follows the flow of the Breede River from source (A) to sea (O). The size of each bar is proportionate to the number of fish recorded and each colour represents a different species. Invasive species are marked with an asterisk (*)

Chi-square tests of independence indicated effects of piscivore gape limitation on the length frequencies of the fish assemblage. Whereby, *C. gariepinus* typically attained large body sizes throughout the system (> 450–550 mm) and limited larger sized bass abundance (X^2^ = 318.8, *p* < *0.01*; Figs. 2, 3). In the absence of bass (site D), we observed a wide variety in *C. capensis* size classes with lengths from 50 to 550 mm. However, where *C. capensis* and bass overlap (M), we observed lengths between 151 and 350 mm, while *M. nigricans* and *M. dolomieu* reached sizes < 250 mm and < 350 mm, respectively (X^2^ = 30.4, *p* < *0.01;* Figs. 2, 3). There was no support for facilitative interactions consistent with invasional meltdown between *C. gariepinus* and *C. carpio,* nor evidence of predation control by *Micropterus* spp. on *C. carpio (*Fig. 3). On the other hand, invasive *C. carpio* generally reached small sizes as most fish were <150 mm. Although little to no middle-sized fish were recorded (250–450 mm), there was a small number of large individuals (> 450 mm) in multiple sites with no correlation to either catfish or bass frequency (Fig. 3).

**Fig. 4:**
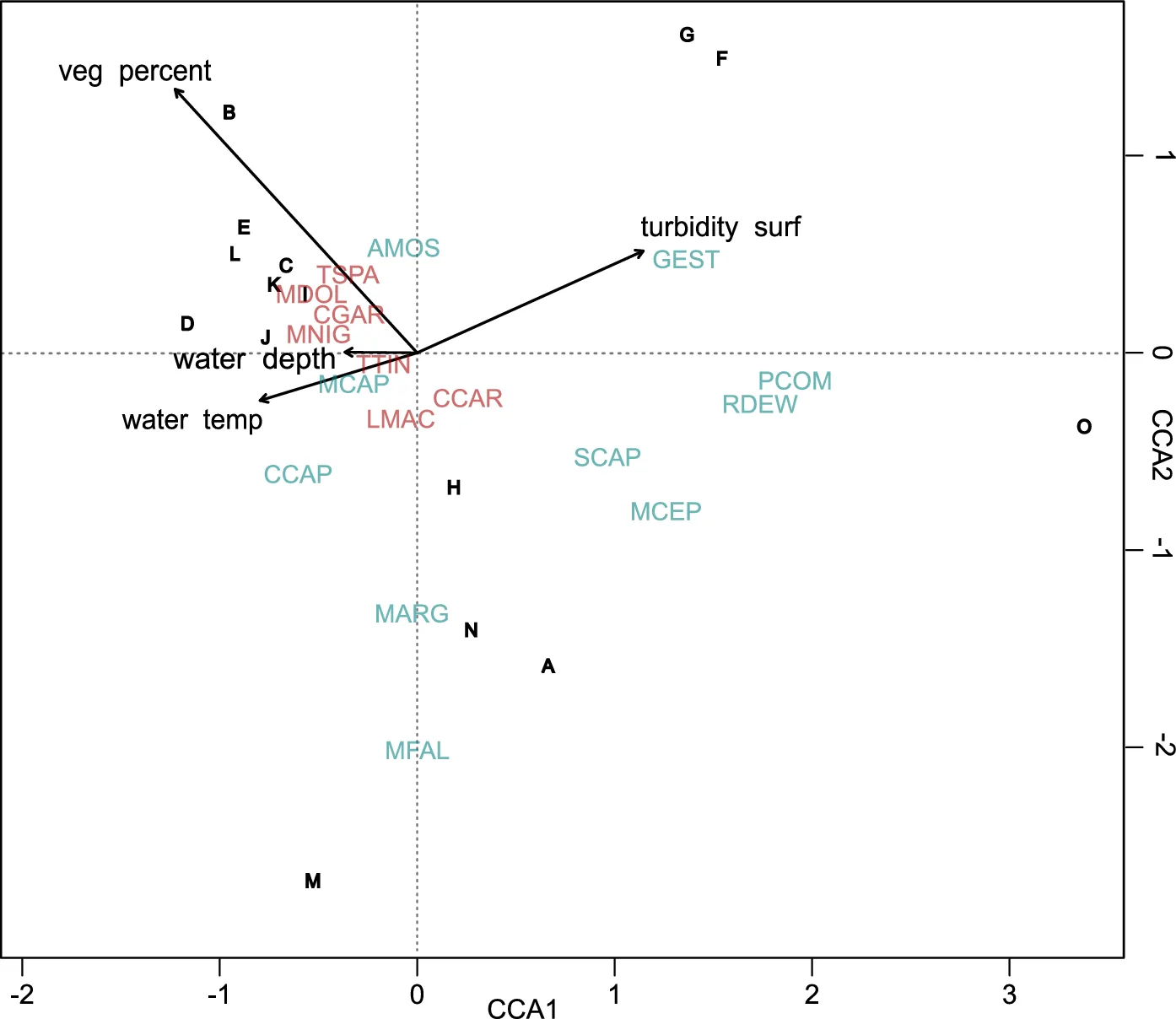
Species diversity along the Breede River is driven by invasive predators and environmental variables. Canonical correspondence analysis (CCA) showing the relationship between species composition and significant environmental variables across different sites in the Breede River. Letters correspond to sampling site codes in alphabetical order: A (source)—O (estuary). veg percent = vegetation coverage %, turbidity surf = below surface water turbidity, water depth = maximum recorded depth, water temp = average water temperature based on surface and bottom measurements. Invasive species are highlighted in red and native species in blue. Species abbreviations: *Anguilla mossambica* (AMOS), *Clarias gariepinus* (CGAR), *Cyprinus carpio* (CCAR), *Gilchrestella aestuaria* (GEST), *Lepomis macrochirus* (LMAC), *Micropterus dolomieu* (MDOL), *Micropterus nigricans* (MNIG), *Monodactylus argenteus* (MARG), *Monodactylus falciformis* (MFAL), *Mugil cephalus* (MCEP), *Myxus capensis* (MCAP), *Pomadasys commersonii* (PCOM), *Cheilobarbus capensis* (CCAP), *Redigobius dewaali* (RDEW), *Sandelia capensis* (SCAP), *Tilapia sparrmanii* (TSPA), *Tinca tinca* (TTIN)

Key habitat characteristics determining species distribution were water turbidity, depth, average temperature, and vegetation coverage (Fig. 4). Turbidity and vegetation coverage combined, explained 72.6% of the observed variance in our dataset and were included as predictors of species frequencies in our models together with the mean human footprint for the areas surrounding each sampling location (Fig. 1). The main predictors of native species frequency were the relative frequency of carp and the mean human footprint index for the surrounding area. Although the positive effect of mean human footprint was not statistically significant and explained limited variation (GLM estimate ± standard error: 0.007 ± 0.005, t_14_= 1.42, *p* = 0.18) it was retained in the model for completeness. Relative frequency of carp had a significantly positive relationship with native species frequency (GLM estimate ± standard error: 0.005 ± 0.002, t_14_= 2.22, *p* < 0.05). Water turbidity, catfish and carp frequencies all had a negative effect on bass frequency. Water turbidity was the main driver of bass distribution across the river with a significant negative effect on relative bass frequency (GLM estimate ± standard error: 0.81 ± 0.08, t_14_= −2.50, *p* < 0.05).

## Discussion

Multiple co-occurring stressors can reshape ecology in an unpredictable fashion. Systems afflicted by numerous invasive species are shaped by the interactions of non-native species on both native and other non-native species. Here we demonstrate how invasive species ecosystem engineering traits can filter out other damaging species and change outcomes for native assemblages. Our results suggest that in a system subjected to the effects of multiple invasive fishes, 1) high densities of sharptooth catfish (*C. gariepinus*) and locally turbid waters potentially associated with carp (*C. carpio*) benthic feeding behaviour are collectively mitigating the impacts of black basses (*Micropterus* spp.) on native prey, whereas 2) the presence of flow barriers potentially limits movement of other invasive species, such as tilapia (*T. sparrmanii*) and tench (*T. tinca*). At the same time, the extremely limited distribution of native species of concern (*S. capensis*, *A. mossambica*, *C. capensis*) is alarming and highlights the need for further assessment and protection measures.

Physical flow barriers, such as dams and weirs, restrict river movement of *M. nigricans* and *M. dolomieu* if of sufficient height, thereby providing refugia for native prey species (Burnett et al. 2023; Van Der Walt et al. 2016). In the Breede River, native *C. capensis* and *S. capensis* are able to persist at low densities in upstream sites where barriers seemingly offer protection from competition with *T. tinca* whose trophic niche overlaps, and predation by large basses. Unlike the highly abundant and impactful predator *C. gariepinus,* the distribution of competitors *T. sparrmanii* and *T. tinca* was limited and concentrated in sites generally restricted between barriers. This pattern is consistent with their low impact on the system but implies their potential to cause broader food-web disruption in the future if the barriers are removed, a common dilemma in freshwater conservation (Dolan et al. 2025). We note that *G. aestuaria* is a euryhaline species typically found in coastal and estuarine habitats (Russell 2011; Whitfield 2025). The presence of this species downstream of the Brandvlei Dam is likely due to a within-catchment range expansion after an undocumented translocation event into the dam. This is similar to dynamics observed in the Eastern Cape, in the Darlington Dam and Sundays River system and demonstrates the readiness at which this species is able to colonise river systems from impoundment introductions (Woodford et al. 2013).

Apart from barriers and other habitat characteristics, it is well established that predation is a key driver of fish community structure (Jackson et al. 2001) and predator–prey interactions between fishes can affect the outcome of non-native fish invasions (Alofs and Jackson 2014; Britton 2023), leading us to anticipate the significant relationship of introduced predators *C. gariepinus, M. nigricans* and *M. dolomieu* with the diversity of native species in the Breede River. When large catfishes are introduced to a system, they tend to outcompete local predators and can shift aquatic food-web dynamics (Hilling et al. 2023; Hodgson et al. 2025; Khan et al. 2021; Low et al. 2022; Weyl et al. 2016). In South Africa, extralimital *C. gariepinus* has demonstrated the ability to successfully establish in varying conditions, despite physical barriers, and threaten a wide range of taxa due to its diverse feeding habits (Cambray 2003; Ellender et al. 2015; Kadye and Booth 2012a, 2012b, 2013). Similarly, predation impacts by black basses on native fish communities are well documented across South Africa (Ellender and Weyl 2014; Khosa et al. 2019; Woodford et al. 2024). They pose a significant threat to endemic cyprinids, such as the Breede River’s *C. capensis,* and are responsible for the extirpation of the threatened minnow *Pseudobarbus afer* in other rivers (Ellender et al. 2011, 2018; Shelton et al. 2017; Van Der Walt et al. 2016; Woodford et al. 2024).

Evidence from other freshwater systems where introduced *C. gariepinus* and bass species *M. nigricans* or *M. dolomieu* co-exist, suggests that *C. gariepinus* has higher invasive potential and can occasionally and partially exclude basses, but *M. nigricans* is still predicted to have a more severe consumptive impact on native biodiversity (Alexander et al. 2014; Ellender et al. 2011, 2015; Khosa et al. 2023; Woodford et al. 2024). Contrary to this prediction, the present study indicates that, in the mainstem of the Breede River, widespread *C. gariepinus* and water turbidity suppress both bass species, resulting in low densities and a smaller impact on fish diversity and endangered *C. capensis*. Specifically, our results suggest *C. capensis* can evade gape-limited predation by surpassing black bass in body size in sites where they are both present. This shows that adult fish can persist despite bass presence, but recruitment success is unlikely due to persistent predation risk for juveniles, thus resulting in a sink population of adults (Woodford and McIntosh 2011; Ellender et al. 2018). Unlike catfishes, basses are predominantly diurnal visual predators (Gardiner and Motta 2012; Mitchem et al. 2019) and are less efficient in turbid waters where low visibility restricts their ability to select and capture prey (Huenemann et al. 2012; Shoup and Wahl 2009). This is confirmed by our observation that high turbidity in parts of the Breede River was a significant predictor of black bass frequency and a major driver of fish community composition together with vegetation coverage. Low water clarity could be associated with habitat disturbance and high densities of invasive omnivore *C. carpio* across the river (Ellender and Weyl 2014; Huser et al. 2022; Peterson et al. 2022). Although highly abundant in the Breede River, carps were mainly recorded at smaller sizes (< 150 mm), a result that supports experimental evidence suggesting that young, medium-sized carps (~ 100–160 mm) have a higher impact on water clarity and chemistry (Chumchal et al. 2005; Nieoczym and Kloskowski 2014).

Invasive fish management in South Africa has historically relied on piscicide application (e.g. Weyl et al. 2014; Dalu et al. 2020) but this is generally not suitable in large rivers with high human activity zones. Simple measures, such as ensuring that in-stream barriers remain to retain *T. tinca* and *T. sparrmanii,* is likely to be effective if paired with clear biosecurity communication by local nature management authorities (e.g. CapeNature) (Dolan et al. 2025). This is only suitable for species of low value for recreational and subsistence fishing, i.e. neither *C. gariepinus* nor *M. nigricans,* whereby there is a conservation—stakeholder conflict, and anglers often proliferate the species throughout a river network (Ellender et al. 2014; Woodford et al. 2017; South et al. 2022). Baited longlines, which select only for *C. gariepinus,* offer a potential solution to reducing population density and supporting the nation’s aspirations for developing inland fisheries and subsistence fishing (Richardson et al. 2009; Barkhuizen and Swanepoel 2025). River rehabilitation and subsequent nature-based solutions may offer a unique way to mitigate the predatory impact of bass. As visual-pursuit predators, bass predatory potential is reduced in complex habitats that offer refugia for prey and support native ambush predators (Ellender et al. 2018; Khosa et al. 2021; Luger et al. 2020). Conservation interventions for threatened native species should be conducted alongside invasive species removals. Focus on ensuring recruitment success and head-starting of *C. capensis* could mirror efforts in the Olifants-Doring system to save the sandfish (*Labeo seeberi*) population that has historically failed to recruit due to bass predation (Cerilla et al. 2022, 2024). Incorporating active management interventions in the Breede River to reduce negative ecological impacts and prevent farther spread is critical. Nonetheless, management decisions need to be carefully designed for each species and with species interactions in mind. This study addresses the literature gap on the dynamics of multi-species invasions in South Africa, where studies have primarily focused on terrestrial ecosystems and invasions on individual species basis (van Wilgen et al. 2022). Our observations in the Breede River generally fit past predictions of invasive fish spread and impact from other South African systems (Ellender and Weyl 2014; Khosa et al. 2019, 2023; Marr et al. 2017) but provide new insights into the antagonistic interactions of co-occurring invasive fishes with environmental parameters and native ichthyofauna. Despite this survey being restricted to a single sampling season in 2016, the results show the value of longitudinal river biodiversity sampling and appraising the effects of biotic and abiotic interactions on assemblages. Repeating such baseline surveys over time and space can help support whether these inferences are consistent and facilitate the use of more nuanced analytical processes, such as joint species distribution modelling or co-interaction network modelling, but this requires ongoing funding and skilled personnel. This is a crucial step in safeguarding the future resilience of endangered species and other similar systems where species introductions and intensifying human presence are becoming a threat to local biodiversity.

## Data Availability

Supporting data are available in the main manuscript. Additional data will be made available on reasonable request.
